# Recurrent cardiac myxoma in a 25 year old male: a DNA study

**DOI:** 10.1186/1477-7819-11-95

**Published:** 2013-04-25

**Authors:** Marina Kontogiorgi, Vasiliki E Kalodimou, George Samanidis, Vasiliki Vartela, Androniki Tasouli, Abraham Ghiatas, Loukas Kaklamanis, Andreas Karabinis, Alkiviadis Michalis

**Affiliations:** 1 Critical Care Department, Onasis Cardiac Surgery Center Hospital, Athens, Greece; 2 Flow Cytometry-Research & Regenerative Medicine Department, IASO Maternity Hospital, Athens, Greece; 3 Cardiovascular Department, Onasis Cardiac Surgery Center Hospital, Athens, Greece; 4 Department of Cardiology Onasis Cardiac Surgery Center Hospital, Athens, Greece; 5 Radiology Department IASO Maternal Hospital, Athens, Greece; 6 Pathology Department Onasis Cardiac Surgery Center, Hospital Athens, Athens, Greece; 7 Critical Care Department, Onasis Cardiac Surgery Center Hospital, 356 Sygrou Avenue, Athens 17654, Greece

**Keywords:** Cardiac myxoma, Aneuploid DNA

## Abstract

We present a 25 year old Caucasian male patient with multiple recurrences of cardiac myxomas after surgical removal of the original tumor. His mother was operated on for right ventricular myxoma. The genetic analyses disclosed an aneuploid DNA content by flow cytometry analysis. The familial form of the cardiac myxomas must be distinguished from Carney complex syndrome. A long- term echocardiographic follow up is recommended to patients and their first degree relatives with cardiac myxomas.

## Background

Primary tumors of the heart are extremely rare with a prevalence rate of around 0.0017% to 0.003% in collective autopsy series. The majority of them are benign [1,2]. Cardiac myxomas are the commonest primary benign tumor in adults. The natural history of sporadic cardiac myxomas is characterized by a predilection for the left atrium of the heart, and occurs in 75% of cases, mainly in middle-aged women and arising usually from the interatrial septum at the border of the fossa ovalis. They have an incidence of relapse in up to 3%, by contrast with familial and complex types of disorder which have recurrence rates 12% and 22% respectively [3].

The familial form of cardiac myxomas is characterized by occurring in a younger patient age group and predominately in males, and with unusual location of the tumor with a tendency to be multifocal in one or more chambers of the heart [2,4]. The association of cardiac myxomas with primary pigmented nodular adrenocortical disease, pituitary-related symptoms and multiple neoplasia syndromes of the cutaneous, thyroid, testicular, breast and neural systems are referred as complex myxomas or Carney syndrome.

The size, mobility and location determine the clinical manifestations of the tumor. The typical triad includes intracardiac obstruction, embolic events and constitutional symptoms. The echocardiography examination is a useful diagnostic tool to determine the tumor size and type, anatomical localization and valvular abnormalities [2,5]. The MRI and CT scan can also often be helpful in clarifying the diagnosis of cardiac myxomas.

In the presence of clinical and /or imaging evidence of a cardiac mass, surgical excision must be performed in order to avoid complications of intracardiac obstruction and embolization of tumor fragments or thrombi. The high incidence of recurrence of the myxoma in our case highlights the difficult management of the tumor and underlines the necessity of the genetic profile analysis in the younger age group patients and their first degree relatives.

## Case presentation

In 2002, a 25 year old Caucasian male patient was referred to Onassis Cardiac Surgery Center with a large (7×6×3 cm) right ventricular myxoma with a stalk attached to the ventricular septum and extending to the chordal apparatus that prolapsed into the right ventricle during systole.

Initially, he presented to the military hospital for routine medical follow-up. Cardiac examination revealed a systolic murmur parasternally on the left. There was no history of chest pain, palpitations, syncope, adrenal or thyroid abnormalities. Pigmentation of derma was not revealed. His family history was positive for myxomas. His mother underwent a surgical resection of a right ventricle myxoma in 1990. She remains asymptomatic.

**Figure 1:**
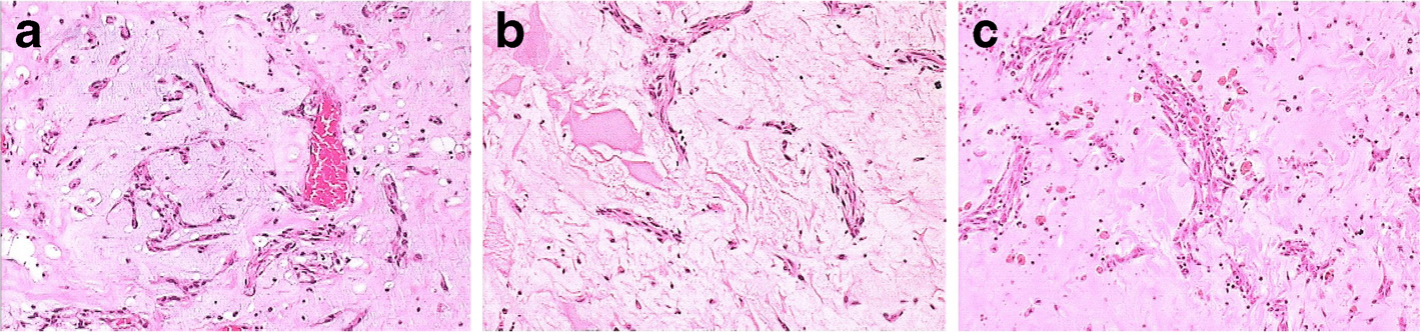
**(a): Right ventricular myxoma with stalk attached to ventricular septum (7×6×3 cm).** Cells were stained with Haematoxylin and Eosin and visualized at × 100 magnification (H+E)x100. (**b**): Right atrial myxoma (5.0×5.0 cm) arising from atrial septum (H+E)×100. (**c**): Left atrial myxoma (2.7 cm) (H+E)×100.

An uncomplicated surgical removal was performed and he was discharge few days later. Histological, the diagnosis of cardiac myxoma was confirmed (Figure 1a).

In 2005, three years after initial excision during a routine follow- up echocardiography, a denticulate lesion of the right atrium (5.0×5.0 cm) arising from atrial septum was disclosed. During this period, he was asymptomatic and he worked without any complaint.

Focusing on positive family history and relapse of the disorder, an evaluation of the presence of Carney complex was conducted. There was none association with a primary pigmented adrenocortical disease, skin lesions or thyroid dysfunction. He underwent a further uneventful surgical resection (Figure 1b).

He had been asymptomatic since 2008, when a new recurrence was revealed. The tumor (4.5×3.5 cm) was located on the posterior wall of the right ventricle under the posterior leaflet of the pulmonary valve. A new operative resection of the mass was performed. Histologically, a myxomatous tissue was identified.

In 2009, he presented with a right hemiparesis, transmissional aphasia and epilepsy. He was transferred to a tertiary hospital and was intubated. The cerebral CT scan disclosed an ischemic lesion of the right hemisphere.

Two- dimensional echocardiography disclosed a tumor (2.7 cm) located in the left atrium next to the origin of right inferior pulmonary vein. After significant improvement of his neurological status, a fourth reoperation was performed with excision of the mass accompanied by patch replacement of pericardium (Figure 1c).

**Figure 2:**
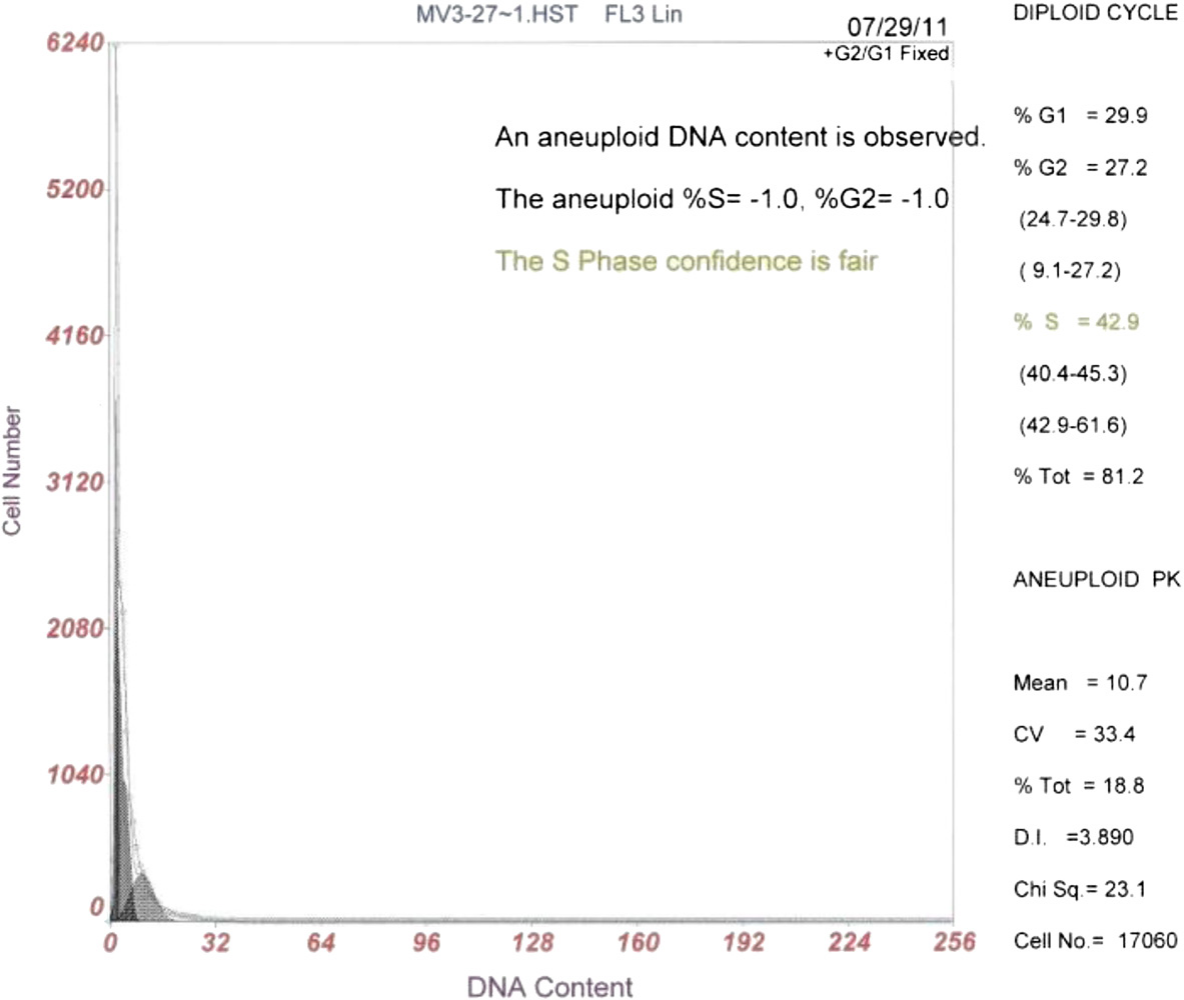
An aneuploid DNA content of cardiac myxoma is revealed after Flow Cytometry analysis.

The postoperative period was uneventful and he was discharged. Samples of the tumor located in the left atrium were analyzed on a Cytomics FC 500 flow cytometer (Beckman Coulter, Leriva company, Athens, Greece), set up with an argon-ion laser emitting at 488 nm (400 m W) for aneuploidy. A minimum number of 17,000 nuclei from each specimen were analyzed. The data were displayed as histograms. The flow cytometry analysis revealed an aneuploid DNA content in the tissue sample (Figure 2).

**Figure 3:**
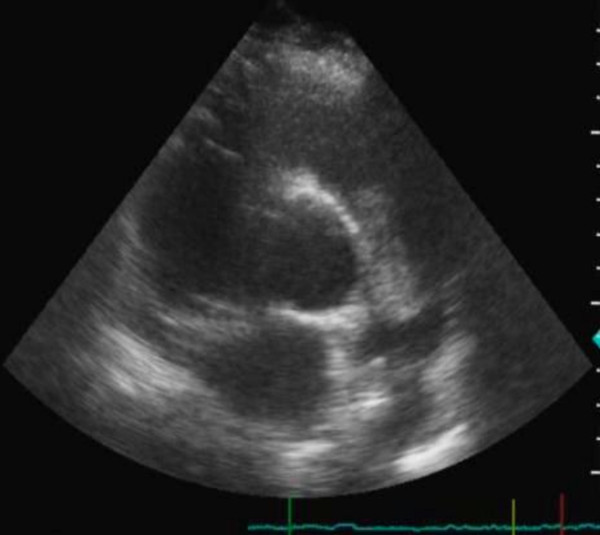
Two-dimensional short-axis view visualizing the left ventricular myxoma and the enlarged right ventricle.

**Figure 4:**
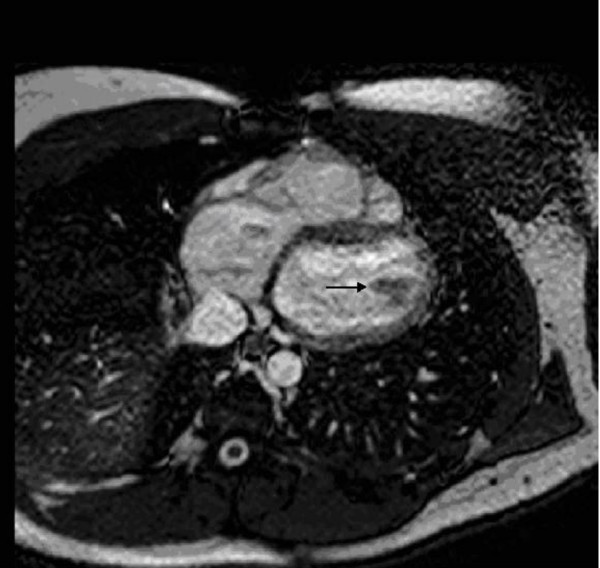
Cardiac MRI: T1WI, with contrast, demonstrates low intensity mass presenting the myxoma.

A year later a new relapse occurred and a left ventricular myxoma was visualized by two- dimensional echocardiography (Figure 3). The echo findings were confirmed by cardiac magnetic resonance (Figure 4). The patient declined to undergo surgical treatment. A conservative approach was agreed with a regular echocardiographic evaluation of the tumor.

## Discussion

Primary tumors of heart are rare, with an incidence ranging from 0.0017% to 0.03% in reported autopsy series, and the majority are benign [1,2]. Cardiac myxoma is the most common primary benign cardiac tumor in adults. It is seen more commonly in females with a wide age range from the third to sixth decade [1]. The clinical features and familial history of patients with cardiac myxomas reveal the differences between sporadic and familial cases.

The appearance of a single tumor in the left atrium of middle-aged women, without other coincidental extracardiac manifestations is associated with non- familial or sporadic type of the disorder. On the other hand, the presence of a tumor in the right atrium in the younger age groups with familial predisposition and high incidence of recurrence is considered to be familial cardiac myxoma [6].

Pateras *et al.* consider that *Herpes simplex virus (HSV)* DNA infection is involved in the pathogenesis of sporadic atrial myxoma. The authors note that the presence of *HSV-1* and/or −2 DNA was detected in 35% of the informative sporadic cardiac myxomas. Furthermore, they consider that the tropism of the virus for myxomatous endocardium and the viral life cycle remain an issue to be addressed concerning the rate of recurrences of the tumor [7].

Stratakis claims that, one-fifth of patients with familial myxomas present with extracardiac involvement consisting of a primary pigmented nodular adrenocortical disease, skin lesions - including lentigines, ephelides, blue nevi, cutaneous myxomas, myxoid mammary fibroadenomas, pituitary adenomas, thyroid and testicular tumors and melanotic Schwannomas. At least, two or more of these findings must be present in order to be diagnostic [4].

Seventy-five percent of the myxomas are located in the left atrium, and 20% in the right atrium, whereas ventricular origination is the most infrequent [8]. According to Pinede *et al.*, the location, size, and mobility of the tumor are related to the clinical symptoms. The cardiac myxomas are located mainly in the left atrium, originating from the atrial septum next to the margin of fossa ovalis. This predilection for the left heart often leads to left-sided congestive heart failure following complete mitral valve obstruction or coronary artery embolism, and systemic embolization occurring in 40% of patients due to small tumor particles or thrombus on the tumor surface [3].

The cardiac myxomas of the right atrium often lead to right-sided congestive heart failure related to venae cavae obstruction and tricuspid valve disease [8]. The presenting symptoms usually are dyspnea, chest pain, syncope, pulmonary hypertension, and cardiac arrhythmias, however, these symptoms are common in cardiac patients generally. Fever, malaise, weight loss, polymyositis, hepatic dysfunction, Raynaud’s phenomenon, hyperglobulinaemia and arthralgias, are also described in patients with atrial myxomas and attributed to an autoimmune response or a result of a secondary tumor infection [9]. The malignant potential of atrial myxomas remains a matter of debate. Few cases of atrial myxomas with extracardiac metastasis have been described in the literature. On the other hand, some authors believe that chondrosarcomas simulate malignant atrial myxomas [8].

The echocardiographic appearance is a useful diagnostic tool in early diagnosis, with high sensitivity. The transesophageal and transthoracic echocardiography (TTE) are able to determine tumor size and type (benign or malignant), anatomical localization and valvular abnormalities [2,5]. CT and MRI have excellent diagnostic advantages regarding tumor delineation and spread. More specifically, MRI currently appears to be the imaging modality of choice in differentiating left atrial myxoma from malignancies [10].

A long term follow-up is recommended to patients and their first degree siblings, especially, with familial cardiac myxomas. Prognosis is usually favorable with considerable potential for full recovery. However, lethal outcome in unrecognized and untreated cases can also occur due to coronary or cerebral embolization and complete obstruction of mitral or tricuspid valve. Also, the prognosis for patients with Carney syndrome is reserved due to the malignant potential of pituitary, testicular, thyroid and metastasizing pancreatic tumors or melanotic Schwannomas [3].

The main treatment strategy is surgical resection. In the literature, the recurrence rate is between 1 to 3% for sporadic cases of cardiac myxomas and is increased significantly for patients with familial and Carney complex myxomas, at 12% and 22% respectively [3]. Some authors suggest that treatment with suppressive anti - *HSV* drugs such as acyclovir for *HSV* - positive patients postoperatively is beneficial in reducing the rate of recurrences [7]. We consider that anticoagulation treatment in inoperable patients protects against thromboembolic events.

The high incidence of recurrence results for several reasons: incomplete excision of the tumor, implantation of tumor fragments and familial predisposition [3]. An autosomal dominant inheritance of familial and Carney complex is well- documented. Half of the known Carney complex myxomas can be attributed to mutations in *PRKAR1A* genes [4].

The performed genetic analysis of our patient disclosed an aneuploid DNA (diploid) content (S = −1.0% and G2 = −1.0%), with 42.9% of cells in the DNA synthetic phase confirming the above assumption. DNA analysis by flow cytometry provides fast results, permits multiparameter analysis, correlating DNA content with antigen expression, and provides the sensitivity for detecting near-diploid aneuploid peaks [11]. Unfortunately, we were not able to obtain our patient’s mother’s biopsies in order to conducted genetic analysis for aneuploidy. Finally, in our case, no potential histopathological differences of the tumor were observed among recurrences.

The role of heart transplantation and auto-transplantation remains unclear and requires additional studies [1,2,12,13].

## Conclusion

Cardiac myxoma is very rare benign tumor which can be complicated by extracardiac manifestations leading to disability and death. The diagnosis of the tumor is based on clinical presentation and physical examination confirmed by echocardiography and CT or MRI findings. Natural history of cardiac myxoma requires long - term echocardiographic follow- up of patients and their first degree relatives. High incidence of recurrence is observed in the younger age group, with familial predisposition. The unusual location of the tumor, the tendency for it to be multifocal in one or more chambers of the heart, especially in the younger age groups, gives rise to suspicion of the familial form of cardiac myxomas. In these cases, we consider that a genetic profile analysis should be performed to patients and their first degree relatives.

## Consent

Written informed consent was obtained from the patient for publication of this report and any accompanying images.

## Abbreviation

TTE: transthoracic echocardiography.

## Competing interests

All authors have made substantive contributions to the study, and all authors endorse the conclusion. None of the authors has a conflict of interest.

## Authors’ contribution

KM, TA, KA were involved with the patient’s management. KEV is the head of Critical Care Department, Onasis Cardiac Surgery Center Hospital. KVE is the head of Flow Cytometry-Research & Regenerative Medicine Department, IASO Maternity Hospital, who carried out the DNA analysis. VV is a cardiologist who carried out the echocardiography study. GA is the head of Radiology Department IASO Maternal Hospital and provided the patient’s cardiac MRI. KL is the head of Pathology Department, who carried out histopathology analyses and helped to revision. SG, MA were the patient’s surgeons. MA is the head of Cardiovascular Department, Onasis Cardiac Surgery Center Hospital. KM and KEV prepared, wrote and revised the article. All authors read and approved the final manuscript.
